# *Bacillus subtilis* Induces Human Beta Defensin-2 Through its Lipoproteins in Human Intestinal Epithelial Cells

**DOI:** 10.1007/s12602-024-10224-4

**Published:** 2024-02-20

**Authors:** Yoon Ju So, Ok-Jin Park, Yeongkag Kwon, Jintaek Im, Dongwook Lee, Sung-Ho Yun, Kun Cho, Cheol-Heui Yun, Seung Hyun Han

**Affiliations:** 1 Department of Oral Microbiology and Immunology, and Dental Research Institute, School of Dentistry, Seoul National University, Seoul, 08826 Republic of Korea; 2 Center for Research Equipment, Korea Basic Science Institute, Ochang, 28119 Republic of Korea; 3 Department of Agricultural Biotechnology, and Research Institute of Agriculture and Life Sciences, Seoul National University, Seoul, 08826 Republic of Korea; 4 Institutes of Green Bio Science & Technology, Seoul National University, Pyeongchang, 25354 Republic of Korea

**Keywords:** *Bacillus subtilis*, Lipoprotein, Intestinal epithelial cell, Human beta defensin-2

## Abstract

Human intestinal epithelial cells (IECs) play an important role in maintaining gut homeostasis by producing antimicrobial peptides (AMPs). *Bacillus subtilis*, a commensal bacterium, is considered a probiotic. Although its protective effects on intestinal health are widely reported, the key component of *B. subtilis* responsible for its beneficial effects remains elusive. In this study, we tried to identify the key molecules responsible for *B. subtilis*-induced AMPs and their molecular mechanisms in a human IEC line, Caco-2. *B. subtilis* increased human beta defensin (HBD)-2 mRNA expression in a dose- and time-dependent manner. Among the *B. subtilis* microbe-associated molecular patterns, lipoprotein (LPP) substantially increased the mRNA expression and protein production of HBD-2, whereas lipoteichoic acid and peptidoglycan did not show such effects. Those results were confirmed in primary human IECs. In addition, both LPP recognition and HBD-2 secretion mainly took place on the apical side of fully differentiated and polarized Caco-2 cells through Toll-like receptor 2-mediated JNK/p38 MAP kinase/AP-1 and NF-κB pathways. HBD-2 efficiently inhibited the growth of the intestinal pathogens *Staphylococcus aureus* and *Bacillus cereus*. Furthermore, LPPs pre-incubated with lipase or proteinase K decreased LPP-induced HBD-2 expression, suggesting that the lipid and protein moieties of LPP are crucial for HBD-2 expression. Q Exactive Plus mass spectrometry identified 35 *B. subtilis* LPP candidates within the LPP preparation, and most of them were ABC transporters. Taken together, these results suggest that *B. subtilis* promotes HBD-2 secretion in human IECs mainly with its LPPs, which might enhance the protection from intestinal pathogens.

## Introduction

The human gastrointestinal (GI) tract is home to a diverse abundance of microorganisms [1]. Various cells in the human GI tract, including epithelial, mesenchymal, endothelial, and immune cells, interact directly and indirectly with the gut microbiota and maintain the host-commensal microbial balance by regulating immune responses [2]. Among those diverse cell types, intestinal epithelial cells (IECs), including intestinal epithelial stem cells, Paneth cells, and goblet cells, provide a biochemical and physical barrier that separates commensal bacteria from host cells [3]. IECs interact with intestinal microbes through pattern-recognition receptors such as Toll-like receptors (TLRs) and nucleotide-binding oligomerization domain (NOD)-like receptors, and the expression of those receptors differs along the length of the intestine [4]. The recognition of bacterial microbe-associated molecular patterns (MAMPs) by TLRs triggers downstream signaling pathways, including the mitogen-activated protein (MAP) kinase pathway and nuclear factor kappa B (NF-κB) activation, to regulate gut homeostasis [5].

IECs secrete multiple antimicrobial peptides (AMPs) to maintain mucosal immunity and their amounts can be upregulated upon bacterial infection [6]. AMPs can directly eliminate pathogens by permeabilizing the bacterial membrane and thereby protect the host [7]. So far, four human beta-defensins (HBDs) have been identified as epithelial cell-derived AMPs: HBD-1, HBD-2, HBD-3, and HBD-4 [8]. Among them, HBD-1, HBD-2, and HBD-3 are known to be expressed in the epithelium of the GI tract [9]. Notably, it has been largely accepted that HBD-1 is constitutively expressed, whereas the expression of HBD-2 and HBD-3 is promptly induced by cytokines and microbial infections [10]. Specifically, HBD-2 is considered to be one of the most important AMPs because it can contribute to epithelium integrity, the chemotaxis of immune cells, and the elimination of enteric pathogens [11]. Moreover, it has been reported that dysregulation of HBD-2 is associated with intestinal bowel diseases [12], suggesting its critical role in maintaining gut homeostasis.

*Bacillus subtilis* is an aerobic Gram-positive bacterium found in fermented foods such as natto, soybean pastes, and various fermented soybean products [13]. Since its approval by the Food and Drug Administration as a generally recognized as safe bacteria [14], it has been widely used in the foods, cosmetic, and pharmaceutical industries [15]. It is considered as a probiotic, playing protective roles in intestinal barrier functions. For example, *B. subtilis* can strengthen intestinal barrier functions by increasing the expression of tight junction proteins [16] and downregulating pro-inflammatory cytokines, which can impair intestinal homeostasis when their levels are too high [17]. Oral administration of *B. subtilis* promotes the differentiation of intestinal stem cells into intestinal secretory cells [18] and ameliorates *Salmonella*-induced intestinal disease and dextran sodium sulfate-induced colitis [19]. Furthermore, *B. subtilis* has shown antibacterial, antiviral, and anticancer abilities by producing several compounds, such as cyclic lipopeptides and bacteriocins [20]. Although many studies have focused on the protective roles that *B. subtilis* plays in the intestinal epithelium, the effector molecules responsible for AMP upregulation in humans are poorly understood. Therefore, in this study, we (i) investigated the effects of *B. subtilis* on AMP production and (ii) sought to identify the major cell wall component responsible for AMP induction in human IECs.

## Materials and Methods

### Reagents and Chemicals

*B. subtilis* ATCC 6633 was purchased from the American Type Culture Collection (ATCC; Manassas, VA, USA). *B. subtilis* KCTC 6633, KCTC 3014, KCTC 3135, KCTC 3239, and *Bacillus cereus* KCTC 13153 were purchased from the Korean Collection for Type culture (KCTC; Daejeon, Korea). *Staphylococcus aureus* USA300 was obtained from the Nebraska Transposon Mutant Library (Omaha, NE, USA). DNase I was purchased from Roche Molecular Biochemicals (Laval, QC, Canada). Thiazolyl blue tetrazolium bromide (MTT reagent), JNK V inhibitor, proteinase K, and lipoprotein (LPP) lipase from *Pseudomonas* sp. were obtained from Sigma-Aldrich (St. Louis, MO, USA). Anti-human TLR2 antibody and its isotype control antibody were purchased from Invivogen (San Diego, CA, USA). The inhibitors of ERK (PD98059), JNK (JNK inhibitor V), and p38 MAP kinase (SB203580) were purchased from Calbiochem (San Diego, CA, USA). APC anti-human TLR2 antibody and its isotype control antibody were purchased from Biolegend (San Diego, CA, USA). T-5224, an AP-1 inhibitor, was obtained from ApexBio Technology (Boston, MA, USA), and BAY11-7082, a NF-κB inhibitor, was obtained from Santa Cruz Biotechnology (Santa Cruz, CA, USA). Tryptic soy broth (TSB) was purchased from BD Biosciences (San Diego, CA, USA). All other materials were from Sigma-Aldrich unless stated otherwise.

### Cell Culture

The human epithelial cell line, Caco-2, was obtained from the ATCC. The cells were maintained in complete Dulbecco’s modified Eagle medium (DMEM; Welgene, Daegu, Republic of Korea) with 10% fetal bovine serum (FBS; GIBCO, Burlington, ON, Canada) and 1% penicillin–streptomycin (Hyclone, Logan, UT, USA) at 37℃ in a humidified 5% CO_2_ incubator. For differentiation/polarization, Caco-2 cells were plated on 12-mm transwell inserts with a 0.4-μm pore polycarbonate membrane (Costar, Corning, NY, USA) and incubated for up to 21 days. The differentiation/polarization was confirmed by measuring the trans-epithelial electrical resistance (TEER) at > 400 Ω·cm^2^ with an EVOM2 (World Precision Instruments, Sarasota, FL, USA). Primary human IECs, SNU-61 and SNU-407 cells, were obtained from the Korean Cell Line Bank (Seoul, Republic of Korea) and cultured in complete Roswell Park Memorial Institute-1640 medium (Welgene) containing 10% FBS and 1% penicillin–streptomycin at 37℃ in a humidified CO_2_ incubator.

### Preparation of Heat-Killed Bacteria

*B. subtilis* ATCC 6633 was grown in TSB medium at 37°C in a shaking condition until they reached mid-log phase. Bacteria were harvested by centrifugation at 6200 × *g* (8000 rpm) for 10 min, and bacterial pellets were washed with phosphate-buffered saline (PBS) and incubated at 70°C for 2 h. To confirm that all the bacteria were killed, the heat-killed bacteria were plated on a TSB agar plate (TSB broth containing 1.5% Bactoagar) for 24 h. No bacterial colony was observed (data not shown).

### Purification of Lipoteichoic Acid (LTA)

Bacterial pellets of *B. subtilis* ATCC 6633 were harvested by centrifugation at 6,200 × *g* (8,000 rpm) for 10 min at 4°C and washed with PBS (pH 7.0). LTA was isolated as previously described [21]. The bacterial pellets of *B. subtilis* were resuspended in 0.1 M sodium citrate buffer (pH 4.7) and disrupted using ultrasonication for 2 h at a frequency of 20 kHz with stirring. Subsequently, the bacterial lysates were mixed vigorously with an equal volume of n-butanol and the aqueous phase was collected by centrifugation at 10,075 × *g* (13,000 rpm) for 15 min at room temperature. The collected aqueous phase was dialyzed using a semi-permeable dialysis membrane of 1-kDa molecular cutoff (Spectrum Laboratories, Rancho Domingueguz, CA, USA) in endotoxin-free distilled water (Daihan Pahrm. Co. Ltd., Seoul, Korea). Following the dialysis process, the extract was prepared with a 15% n-propanol concentration in 0.1 M sodium acetate buffer and subjected to hydrophobic interaction chromatography using an octyl-Sepharose column (GE Healthcare, Chicago, IL, USA) to obtain fractions containing LTA. Unbound substances were removed through washing with 20% n-propanol in 0.1 M sodium acetate buffer, followed by the elution of LTA-containing fractions in 35% n-propanol with 0.1 M sodium acetate buffer using a fraction collector (Bio-Rad, Hercules, CA, USA). Then, the column fractions containing phosphates were consolidated, dialyzed, and prepared with 30% n-propanol in 0.1 M sodium acetate buffer for an ion-exchange chromatography with DEAE-Sepharose (Sigma-Aldrich). The fractions were subsequently eluted using a linear salt gradient ranging from 0 to 1 M NaCl in the equilibration buffer. The LTA-containing fractions were pooled, dialyzed, and subjected to lyophilization under vacuum (5 Torr; 24 h). The isolated LTA was quantified by measuring its dry weight, and experimental dose was established according to the previous study [22].

### Purification of Peptidoglycan (PGN)

PGN from *B. subtilis* ATCC 6633 was isolated as previously described [23]. Briefly, bacterial pellets were disrupted by a bead beater and then centrifuged to remove cell debris. The supernatants were recentrifuged and the pellets were incubated with 0.5% sodium dodecyl sulfate (SDS) at 60℃ for 30 min to remove proteins. After being washed with PBS, the insoluble PGN was treated with DNase I and RNase at 37℃ for 2 h and then incubated with trypsin at 37℃ for 18 h. The PGN was incubated with 5% trichloroacetic acid (Sigma-Aldrich) at 26℃ for 18 h and then centrifuged. After being washed with distilled water, the pellet was treated with cold acetone to remove LTA. The final pellets were suspended in distilled water.

### Purification of LPP

LPPs from the *B. subtilis* were isolated as described previously [24]. Briefly, bacterial pellets were resuspended in Tris-buffered saline (TBS) (pH 7.4) containing protease inhibitors (2 mM PMSF, 10 μg/ml leupeptin, and 10 μg/ml aprotinin) and disrupted with ultrasonication. The bacterial lysates were suspended in 2% Triton X-114 in TBS and incubated at 4℃ for 2 h. After centrifugation, bacterial debris was removed, and the supernatant was incubated at 37℃ for phase separation. The lysates were centrifuged at 37℃, and the aqueous phase was discarded. Then, an equal volume of TBS was mixed with the Triton X-114 phase and incubated at 37℃ for 15 min to separate the Triton X-114 phase. After repeating the previous step three times, the Triton X-114 phase was mixed with methanol and incubated at -20℃ overnight for precipitation. The precipitated LPPs were dissolved in 10 mM octyl-β-D-glucopyranoside and quantified using BCA protein assay kits (Pierce, Rockford IL, USA). No endotoxins were detected in the purified LPPs (data not shown).

### Real-Time Reverse Transcription-Polymerase Chain Reaction (Real-Time RT-PCR)

Real-time RT-PCR was conducted as previously described [25]. Total RNA was isolated from cells using TRIzol reagent (Invitrogen, Carlsbad, CA, USA) according to the manufacturer’s instructions. Relative mRNA expression levels were normalized with glyceraldhyde-3-phosphate dehydrogenase (GAPDH) and assessed with the 2^−ΔΔCT^ method. The primer sequences used in this study are shown in Table 1.

**Table 1:** Primer sequence for real-time RT-PCR

**Gene**		**Sequence (5′ → 3′)**
*HBD-1*	Forward	GGGCACCCCTACAAAAGGAA
	Reverse	TGGCAAAATGGAAGATGCTAGTC
*HBD-2*	Forward	CTTCACTCAGGAGCAGCAAGC
	Reverse	ACACCAGTGCTGTCCTGTGACA
*HBD-3*	Forward	GCCATGAAGTTGCTGACTGC
	Reverse	TGAAGTTGGCGGCTGGTAAT
*GAPDH*	Forward	TGCTACTGACAACGTGGCTT
	Reverse	CCAGGAAAGCTGGGCAACTA

### Enzyme-Linked Immunosorbent Assay (ELISA)

Caco-2 cells were plated on a 96-well plate overnight and treated with the stimuli indicated in the Figs. 2D, 3C and 5C, D. The culture supernatants were collected, and the levels of HBD-2 protein were measured using ELISA kits (PeproTech) according to the manufacturer’s instructions.

### Identification of the LPP

To examine the impurities in the LPP preparation, the purified LPPs were treated with proteinase K (50 μg/ml) or DNase I (50 μg/ml) at 37℃ for 1 h or with heat at 100℃ for 10 min. To inactivate the lipids, LPP was incubated with the lipases (50 μg/ml) at 37℃ for 12 h. Caco-2 cells (3 $\times$ 10^5^ cells/well) were treated with 1 μg/ml of *B. subtilis* LPPs treated with heat, DNase I, LPP lipase, or proteinase K. After treatment for 6 h, total RNA was isolated, and the mRNA expression of HBD-2 was measured using real-time RT-PCR. To identify the LPPs from the *B. subtilis*, the purified LPPs were separated on a 10% SDS–polyacrylamide (PAGE) gel and stained with Coomassie blue (Fig. 7B). The protein content was analyzed using Q Exactive Plus mass spectrometry (Thermo Fisher Scientific Inc., Waltham, MA, USA), and information about the identified proteins was obtained by comparing the peptide sequences with the Subtiwiki database (http://subtiwiki.uni-goettingen.de) [26].

### Flow Cytometry

Caco-2 cells were detached using an enzyme-free cell dissociation solution (Sigma-Aldrich) to prevent the denaturation of surface proteins. After detachment, the cells were washed twice with cold PBS. The cells were stained with APC-conjugated anti-human TLR2 antibody and its isotype control for 30 min on ice and then washed twice with PBS. The stained cells were fixed using 1% paraformaldehyde, and the expression of TLR2 on the cell was analyzed using flow cytometry (FACSVerse, BD Biosciences).

### Measurement of Bacterial Growth

Caco-2 cells were cultured in DMEM without antibiotics and then treated with 1 μg/ml of LPP for 24 h. The culture medium was collected and centrifuged to remove cell debris. The culture supernatant was stored at -80℃ until needed for further experiments. *B. cereus* KCTC13153 and *S. aureus* USA300 were cultured in TSB medium at 37℃ in aerobic conditions. After 18 h, both bacteria were sub-cultured at 1% and plated onto 96-well plates. Various concentrations of supernatant were prepared by twofold serial dilution. The supernatant was administered to both bacteria, and their growth was determined by measuring the optical density at 600 nm with a microplate reader.

### Statistical Analysis

Data are presented as the mean value ± standard deviation from triplicated samples. Experimental groups were compared with an appropriate control group, and statistical analyses were performed with Student’s *t*-test. Statistical significance was set at *P* < 0.05.

## Results

### Both Live and Heat-Killed *B. subtilis* Increase HBD-2 mRNA Expression

**Fig. 1:**
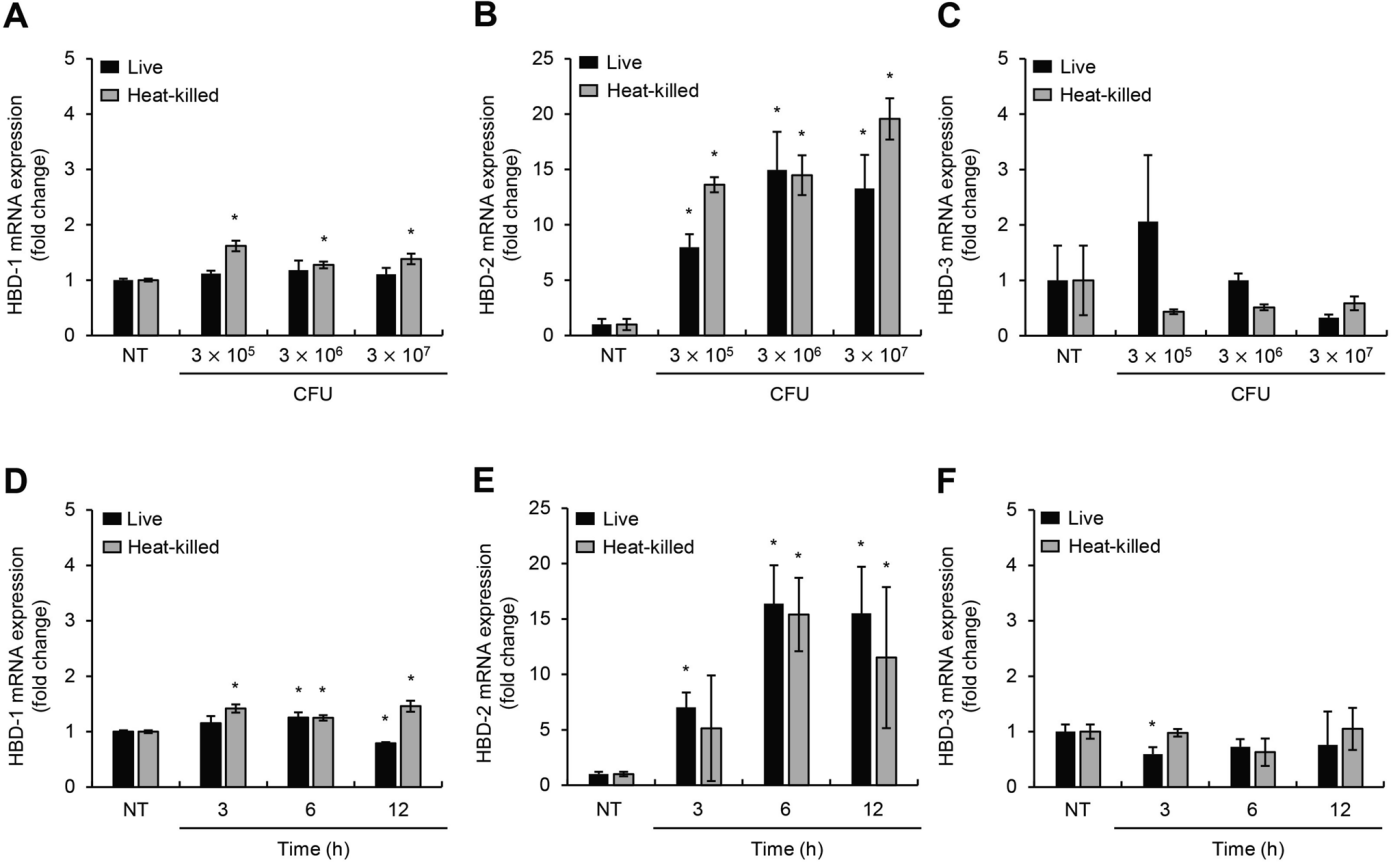
Both live and heat-killed *B. subtills* increase HBD-2 mRNA expression. **A–C** Caco-2 cells (3 $\times$ 10^5^ cells/well) were treated with live- and heat-killed *B. subtilis* (3 $\times$ 10^5^, 3 $\times$ 10^6^, or 3 $\times$ 10^7^ CFU) for 6 h. **D–F** Caco-2 cells (3 $\times$ 10^5^ cells/well) were treated with 3 $\times$ 10^7^ CFU of live- or heat-killed *B. subtilis* for the indicated times. After the treatment, total RNA was isolated and the mRNA expression of AMPs was analyzed using real-time RT-PCR. All results are expressed as the mean $\pm$ standard deviation (S.D.) of triplicate samples. Asterisks (*) indicate statistically significant differences (*P* < 0.05) compared with the appropriate control. NT, non-treatment

To investigate whether *B. subtilis* could induce HBD expression, Caco-2 cells were treated with live or heat-killed *B. subtilis* strain, and then the mRNA expression of HBD-1, HBD-2, and HBD-3 was measured with real-time RT-PCR. As shown in Fig. 1A–C, only HBD-2 mRNA expression was significantly upregulated upon treatment with either live or heat-killed *B. subtilis* in a dose-dependent manner while HBD-1 and HBD-3 expression remained constant. To elucidate the time kinetics of the expression of each AMP, the cells were treated for various times with 3 $\times$ 10^7^ CFU of live or heat-killed *B. subtilis*. HBD-2 mRNA expression was highest at 6 h post treatment in both the live and heat-killed *B. subtilis* treatment groups. However, the expression of HBD-1 and HBD-3 did not change during the course of stimulation (Fig. 1D–F). These results suggest that both live and heat-killed *B. subtilis* induce HBD-2 mRNA expression in human IECs.

### *B. subtilis* LPP Is Responsible for HBD-2 Production in Human IECs

**Fig. 2:**
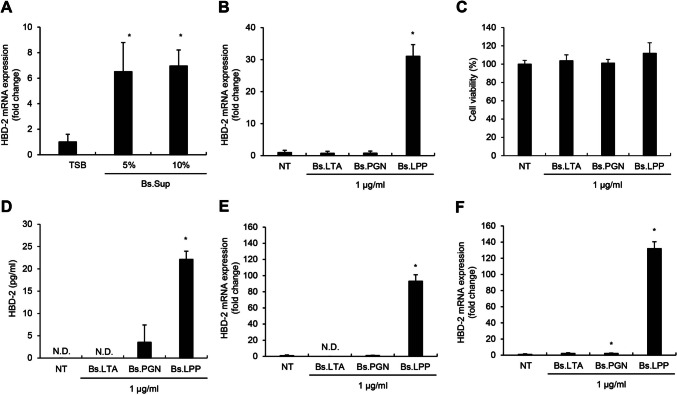
*B. subtilis* lipoprotein (Bs.LPP) significantly induces HBD-2 production in human IECs. Caco-2 cells (3 $\times$ 10^5^ cells/well) were treated with the indicated concentrations of **A** *B. subtilis* culture supernatants (Bs.sup) or **B** 1 μg/ml of *B. subtilis* lipoteichoic acid (Bs.LTA), peptidoglycan (Bs.PGN), or Bs.LPP for 6 h. Total RNA was isolated and mRNA expression of HBD-2 was measured using real-time RT-PCR. **C, D** Caco-2 cells (2 $\times$ 10^5^ cells/well) were plated on a 96-well plate and stimulated with Bs.LTA, Bs.PGN, or Bs.LPP for 24 h. **C** Cell viability was measured using MTT reagent. **D** After the treatment, culture supernatants were collected, and the HBD-2 concentration was measured using ELISA. **E, F** Human primary IECs, **E** SNU-407 cells (3 $\times$ 10^5^ cells/well) and **F** SNU-61 cells (3 $\times$ 10^5^ cells/well), were treated with 1 μg/ml of Bs.LTA, Bs.PGN, or Bs.LPP for 6 h. Total RNA was isolated, and the mRNA expression of HBD-2 was measured by real-time RT-PCR. All results are expressed as the mean $\pm$ standard deviation (S.D.) of triplicate samples. Asterisks (*) indicate statistically significant differences (*P* < 0.05) compared with the appropriate control. TSB, tryptic soy broth; NT, non-treatment; N.D., non-detected

To determine which molecule is responsible for the increase in HBD-2 expression, we collected the culture supernatant of *B. subtilis* and administered it to Caco-2 cells. The culture supernatant increased HBD-2 mRNA expression (Fig. 2A), suggesting that the effector molecules can be released from *B. subtilis*. Because bacterial MAMPs can be released into culture supernatants and are known to have immunoregulatory abilities, it is likely that MAMPs contribute to the upregulation of HBD-2. Therefore, each MAMP (LTA, PGN, or LPP) was isolated from *B. subtilis*, and an equivalent concentration of each one was administered to Caco-2 cells to investigate and compare their HBD-2 induction ability. Interestingly, LPP significantly increased the mRNA expression of HBD-2, whereas the effects of LTA and PGN on HBD-2 expression were negligible (Fig. 2B). In addition, LPP upregulated HBD-2 secretion without affecting the viability of the Caco-2 cells (Fig. 2C, D). These results indicate that LPP is a major molecule responsible for HBD-2 induction in Caco-2 cells. To confirm that the increase in HBD-2 caused by LPP treatment was a general phenomenon, we tested primary human IECs. SNU-407 and SNU-61 cells were treated with *B. subtilis* LTA, PGN, or LPP, and the mRNA expression of HBD-2 was measured by real-time RT-PCR. As shown in the results from Caco-2 cells, LPP treatment produced potent HBD-2 mRNA expression in both primary human IEC lines, whereas LTA and PGN did not show much effect (Fig. 2E, F). These results demonstrate that the induction of HBD-2 by *B. subtilis* LPP is a general phenomenon for human IECs.

### *B. subtilis* LPP Upregulates HBD-2 Expression in Caco-2 Cells

**Fig. 3:**
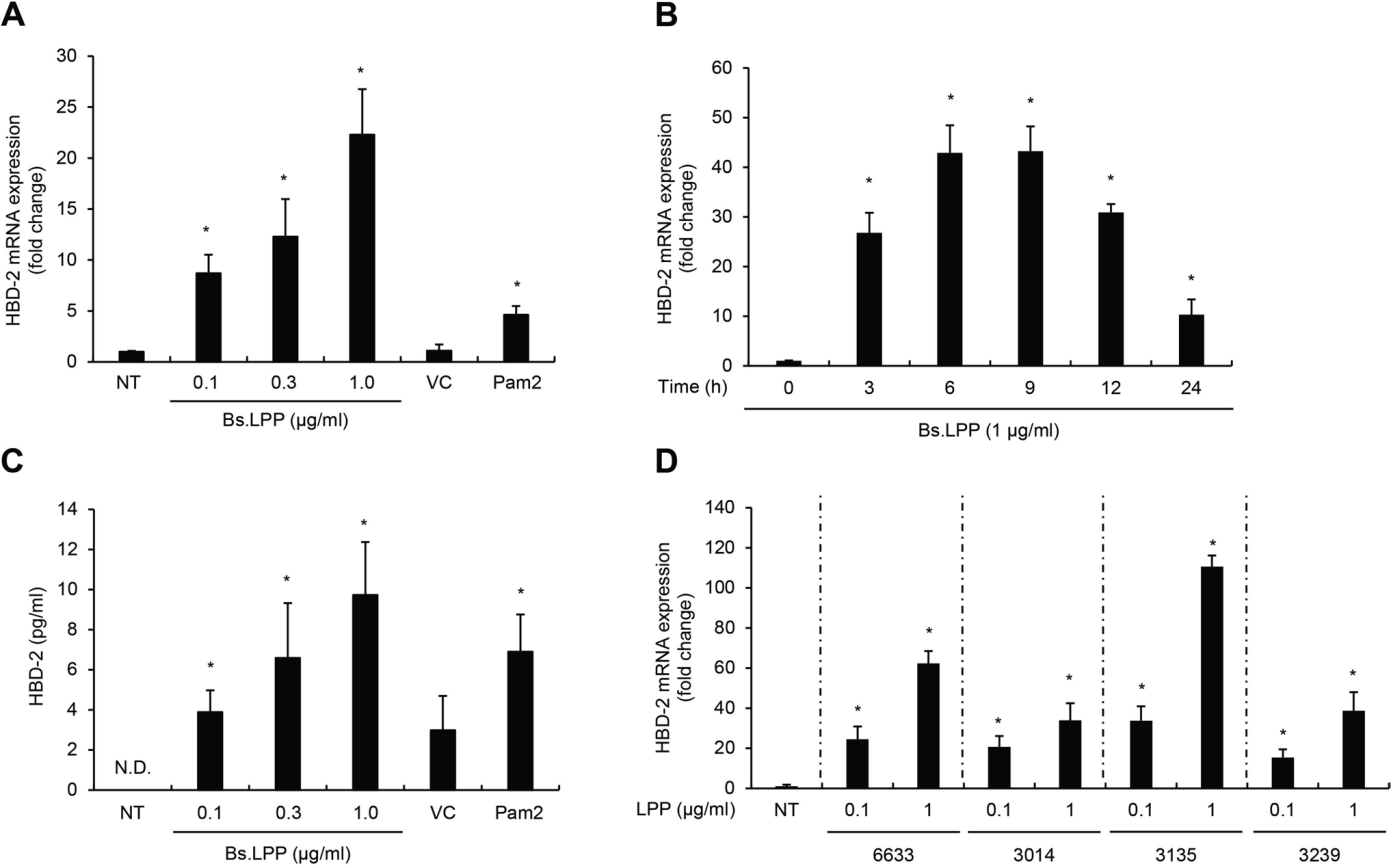
Bs.LPP increases HBD-2 mRNA expression and protein secretion in a dose- and time-dependent manner. **A, B** Caco2 cells (3 $\times$ 10^5^ cells/well) were treated with **A** various doses of Bs.LPP for 6 h or **B** 1 μg/ml of Bs.LPP for the indicated times. Total RNA was isolated, and the mRNA expression of HBD-2 was measured using real-time RT-PCR. **C** Caco-2 cells (2 $\times$ 10^5^ cells/well) were stimulated with various doses of Bs.LPP for 24 h. Culture supernatants were collected, and the HBD-2 concentration was analyzed by ELISA. **D** Caco-2 cells (3 $\times$ 10^5^ cells/well) were treated with 0.1 or 1 μg/ml of LPP from four different KCTC *B. subtilis* strains for 6 h. Total RNA was isolated and the mRNA expression of HBD-2 was analyzed using real-time RT-PCR. All results are expressed as the mean $\pm$ standard deviation (S.D.) of triplicate samples. Asterisks (*) indicate statistically significant differences (*P* < 0.05) compared with the appropriate control. NT, non-treatment; N.D., non-detected; VC, vehicle control; Pam2, Pam2CSK4

To investigate the kinetics of LPP-induced HBD-2 production, Caco-2 cells were stimulated with various concentrations of *B. subtilis* LPP and HBD-2 mRNA expression was measured using real-time RT-PCR. As shown in Fig. 3A, LPP dose-dependently increased HBD-2 mRNA expression in Caco-2 cells. The time kinetics of LPP-induced HBD-2 expression peaked at 6 to 9 h and then decreased by 12 h after the treatment (Fig. 3B). Concordant with the mRNA expression, LPP dose-dependently increased HBD-2 protein secretion in Caco-2 cells (Fig. 3C). These results suggest that LPP from *B. subtilis* upregulates HBD-2 expression in a dose- and time-dependent manner. To examine whether LPPs from other *B. subtilis* strains have the same effect on HBD-2 upregulation, four strains of *B. subtilis*, KCTC 6633, KCTC 3014, KCTC 3135, and KCTC 3239, were obtained and LPPs purified from each strain were administered to Caco-2 cells. All the LPPs tested substantially induced HBD-2 mRNA expression, with LPP from the KCTC 3135 strain inducing the most potent expression of HBD-2 mRNA among the strains tested (Fig. 3D). These results suggest that HBD-2 induction by LPP is a general phenomenon of most *B. subtilis* strains, though the induction rate varies by strain.

### TLR2-Mediated Activation of the JNK/p38 MAP kinase/AP-1 and NF-κB Pathway Is Involved in LPP-Induced HBD-2 Expression

**Fig. 4:**
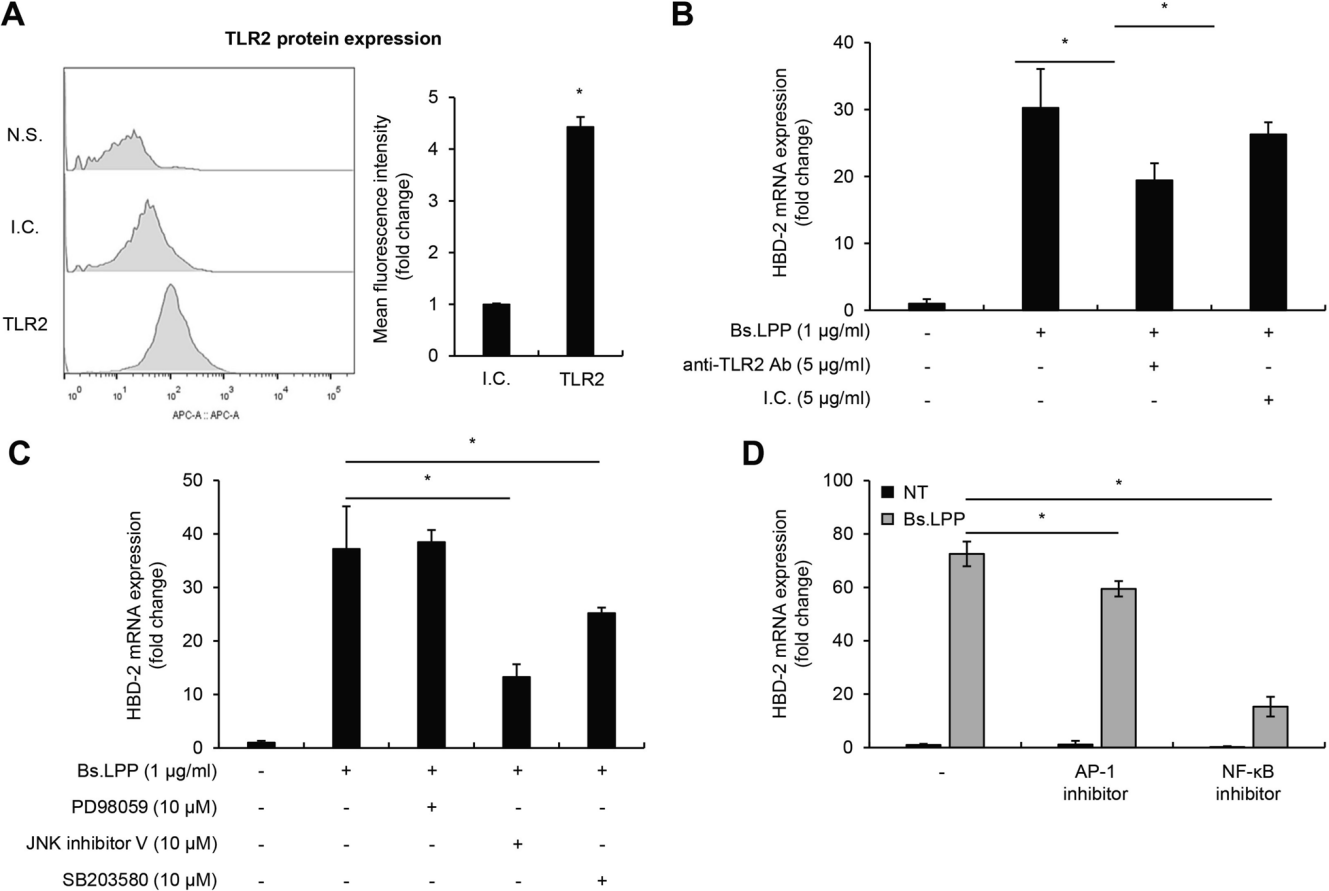
Bs.LPP induces HBD-2 mRNA expression via TLR2-mediated JNK/p38 MAP kinase/AP-1 and NF-κB pathways. **A** Caco-2 cells (3 $\times$ 10^5^ cells) were stained with APC-conjugated anti-human TLR2 (TLR2) or its isotype control (I.C.) antibody. Protein expression of TLR2 was analyzed using flow cytometry. The APC-positive cells are shown as a histogram (left panel), and the ratio of mean fluorescence intensity is shown as a graph (right panel). N.S. indicates the non-staining group. **B** Caco-2 cells (3 $\times$ 10^5^ cells/well) were pre-treated with 5 μg/ml of anti-human TLR2 neutralizing antibody (anti-TLR2 Ab) or its isotype control (I.C.) for 1 h and then stimulated with 1 μg/ml of LPP for 6 h. Caco-2 cells (3 $\times$ 10^5^ cells/well) were pre-treated with **C** MAP kinase inhibitors or **D** an AP-1 inhibitor (T-5224, 40 μM) and NF-κB inhibitor (BAY11-7082, 2.5 μM) for 1 h and then treated with 1 μg/ml of Bs.LPP for 6 h. Total RNA was isolated, and the mRNA expression of HBD-2 was measured by real-time RT-PCR. All results are expressed as the mean $\pm$ standard deviation (S.D.) of triplicate samples. Asterisks (*) indicate statistically significant differences (*P* < 0.05) compared with the appropriate control. NT, non-treatment

Previous studies showed that bacterial LPPs are mainly recognized by TLR2 [27]. Therefore, Caco-2 cells were stained with anti-human TLR2 antibody to examine the expression pattern of TLR2. The result shows that TLR2 is mainly expressed on the cell (Fig. 4A). In addition, to investigate whether the induction of HBD-2 expression by LPP is mediated by a TLR2 signaling pathway, the cells were pre-treated with a TLR2-neutralizing antibody and then stimulated with LPP. Pre-treatment with the anti-TLR2 antibody downregulated the mRNA expression of HBD-2 induced by LPP stimulation, whereas the isotype control antibody did not affect the mRNA expression (Fig. 4B). Therefore, the TLR2 pathway is involved in the upregulation of HBD-2 expression induced by LPP. Furthermore, it has been well documented that TLR2 activation leads to downstream signaling pathways such as MAP kinase and NF-κB translocation to initiate proper immune responses [28]. Therefore, to investigate the intracellular mechanism of HBD-2 induction, Caco-2 cells were pre-treated with several inhibitors and then stimulated with LPP. As shown in Fig. 4C, MAP kinase inhibitors, including JNK inhibitor V (JNK inhibitor) and SB203580 (p38 MAP kinase inhibitor), but not PD98059 (ERK inhibitor), significantly reduced HBD-2 mRNA expression. JNK inhibitor V inhibited the LPP-induced mRNA expression of HBD-2 more than the other MAP kinase pathway inhibitors, suggesting that the JNK pathway is the most important one for the induction of HBD-2 expression. Also, T5224, an AP-1 inhibitor, modestly downregulated HBD-2 mRNA expression, whereas BAY11-7082, an NF-κB inhibitor, dramatically decreased HBD-2 mRNA expression (Fig. 4D). Those results suggest that LPP upregulates HBD-2 expression mainly by means of a JNK/p38 MAP kinase and NF-κB pathways, with the AP-1 pathway only partially involved in HBD-2 induction.

### Differentiated Caco-2 Cells Produce HBD-2 upon *B. subtilis* LPP Treatment

**Fig. 5:**
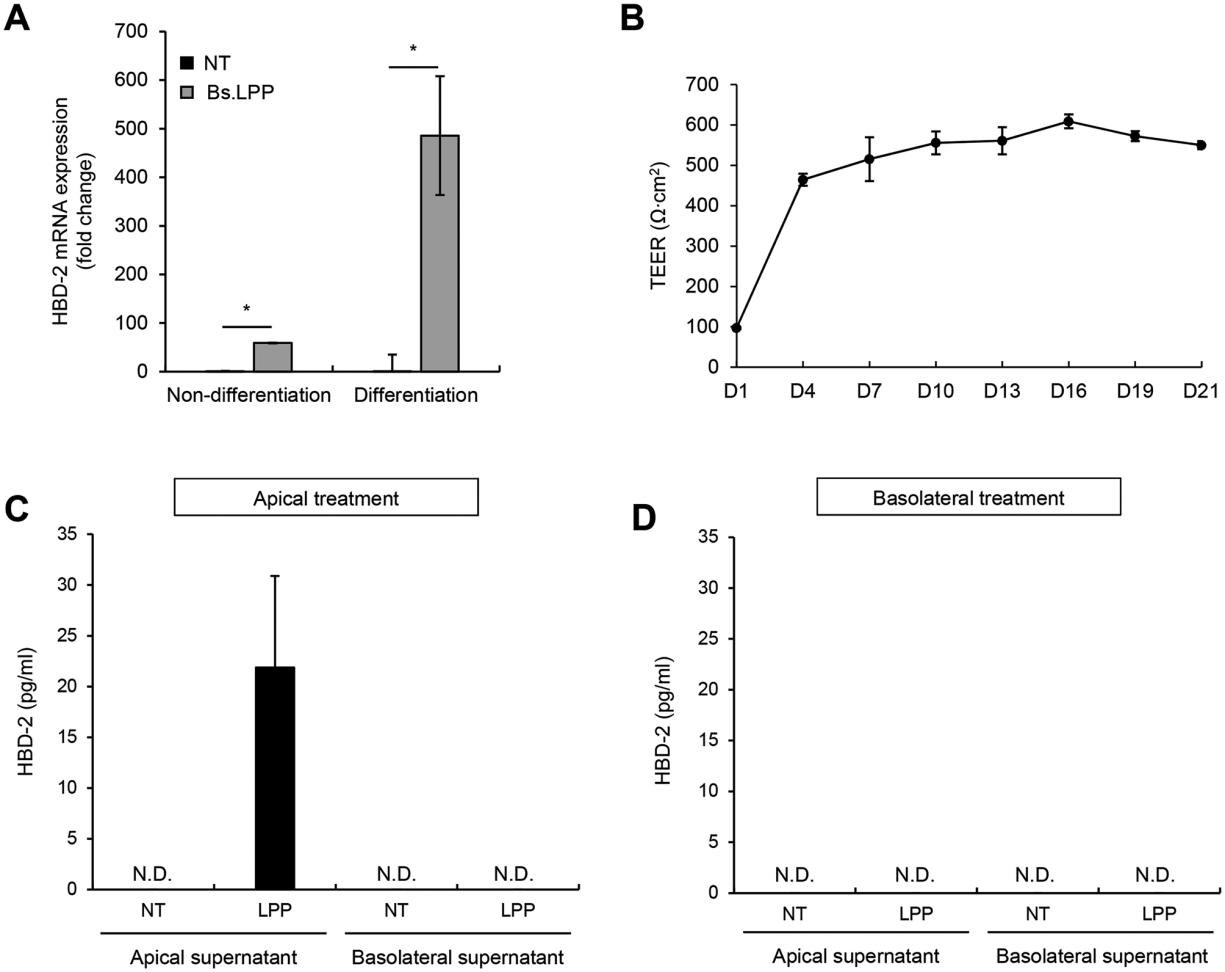
Bs.LPP induces HBD-2 production on the apical side of differentiated Caco-2 cells. **A** Caco-2 cells (3 $\times$ 10^5^ cells/well) were plated on 6-well plates and separated into two groups: non-differentiation and differentiation. The cells of the non-differentiated group were stimulated with 1 μg/ml of Bs.LPP for 6 h. The cells of the differentiation group were cultured for 21 days for polarization, and then the differentiated cells were stimulated with 1 μg/ml of Bs.LPP for 6 h. Total RNA was isolated, and the mRNA expression of HBD-2 was analyzed using real-time RT-PCR. **B–D** Caco-2 cells (1 $\times$ 10^5^ cells/well) were seeded on a transwell plate and cultured for 21 days for differentiation. **B** Transepithelial electrical resistance values were measured for 21 days at three-day intervals using an EVOM2. Differentiated cells were treated with Bs.LPP **C** apically or **D** basolaterally for 24 h. Culture supernatants were collected and the concentration of HBD-2 was measured using ELISA. All results are expressed as the mean $\pm$ standard deviation (S.D.) of triplicate samples. Asterisks (*) indicate statistically significant differences (*P* < 0.05) compared with the appropriate control. NT, non-treatment; N.D., non-detected

It was previously reported that Caco-2 cells can differentiate into cells that morphologically and functionally express the characteristics of mature enterocytes [29]. Therefore, to demonstrate the effect of *B. subtilis* LPPs on HBD-2 gene expression in differentiated IECs, non-differentiated and differentiated Caco-2 cells were treated with LPP and the mRNA expression of HBD-2 was analyzed. Interestingly, the upregulated mRNA expression of HBD-2 after differentiation was about tenfold higher than that in non-differentiated cells (Fig. 5A). To further investigate the effect of *B. subtilis* LPP on HBD-2 protein secretion, Caco-2 cells were cultured in transwell plates for 3 weeks and TEER was measured to confirm monolayer formation and cell polarization. As shown in Fig. 5B, TEER reached a plateau after 4 days of culture and remained constant until 21 days of differentiation, suggesting that the Caco-2 cells were fully differentiated. After differentiation, the cells were treated with LPP apically or basolaterally. Apical LPP treatment increased HBD-2 production in the apical compartment, but HBD-2 was not detected on the basolateral side (Fig. 5C). Interestingly, basolateral treatment of LPP did not induce HBD-2 secretion on either the apical or basolateral compartment (Fig. 5D). Collectively, these results indicate that both LPP recognition and HBD-2 secretion occurred on the apical side of differentiated Caco-2 cells.

### LPP-Induced HBD-2 Inhibits the Growth of Intestinal Pathogens

**Fig. 6:**
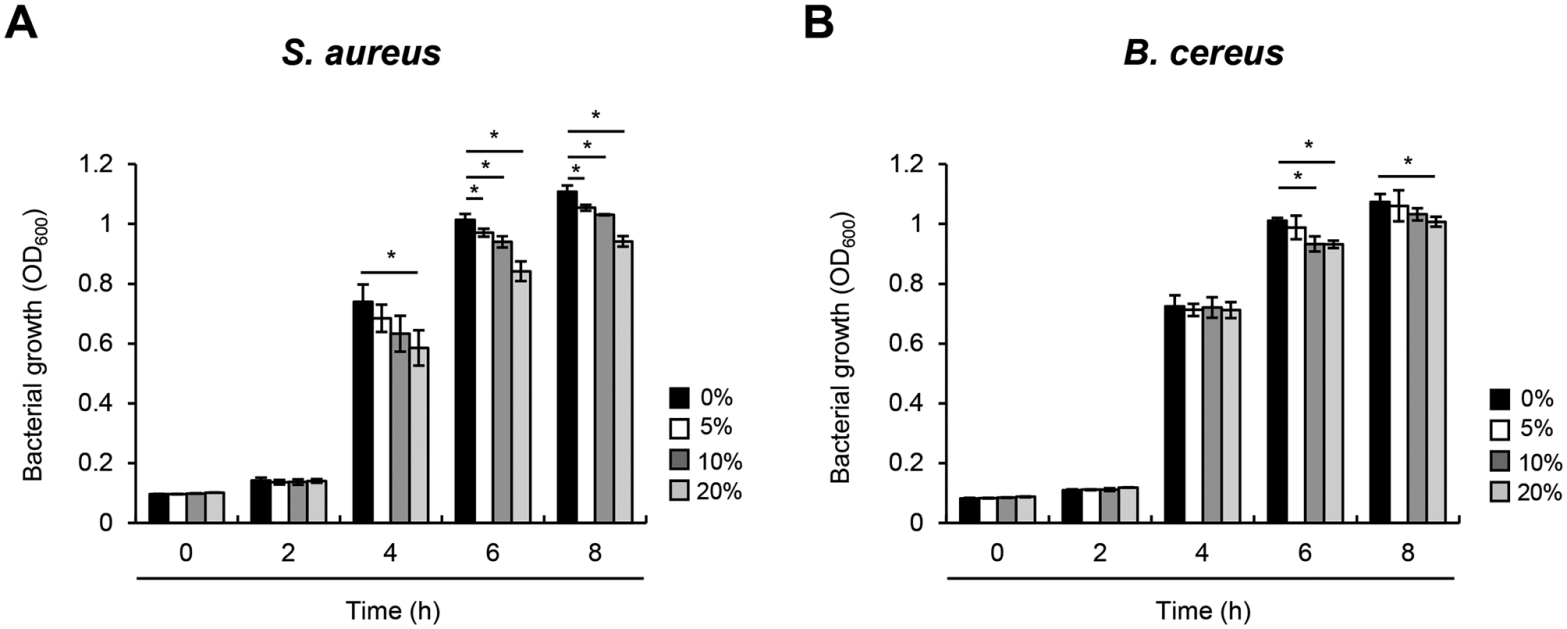
Bs.LPP-induced HBD-2 efficiently inhibits the growth of bacterial pathogens. Caco-2 cells (1 $\times$ 10^6^ cells/well) were stimulated with 1 μg/ml of Bs.LPP for 24 h in antibiotic-free medium. The culture media were collected and centrifuged to remove cell debris. The bacteria, which were sub-cultured at 1%, were plated onto 96-well plates and treated with conditioned media in twofold serial dilutions. The optical density (OD) of **A** *S. aureus* USA300 and **B***. cereus* was measured at each time point using a microplate reader at 600 nm. All results are expressed as the mean $\pm$ standard deviation (S.D.) of triplicate samples. Asterisks (*) indicate statistically significant differences (*P* < 0.05) compared with the appropriate control

It was previously demonstrated that HBD-2 has bactericidal effects against pathogenic bacteria, thereby protecting the host from microbial infection [11]. To examine whether HBD-2 induced by LPP treatment contributes to the elimination of intestinal pathogens, Caco-2 cells were treated with *B. subtilis* LPP for 24 h in antibiotic-free medium and the culture supernatant was collected. The supernatant was diluted with twofold serial dilution and then treated with *B. cereus*, which causes food poisoning [30], or *S. aureus*, which causes secondary GI disorders [31]. The culture supernatant of LPP-treated Caco-2 cells dose-dependently inhibited the growth of both *S. aureus* and *B. cereus.* The culture supernatant of LPP-treated Caco-2 cells reduced the growth of *S. aureus* with a 20% inhibition rate (Fig. 6A) and *B. cereus* with an inhibitory rate of nearly 10% (Fig. 6B). Collectively, these results indicate that HBD-2 secretion induced by LPP treatment of Caco-2 cells inhibits the growth of intestinal pathogens.

### Identification of *B. subtilis* LPP Candidates

**Fig. 7:**
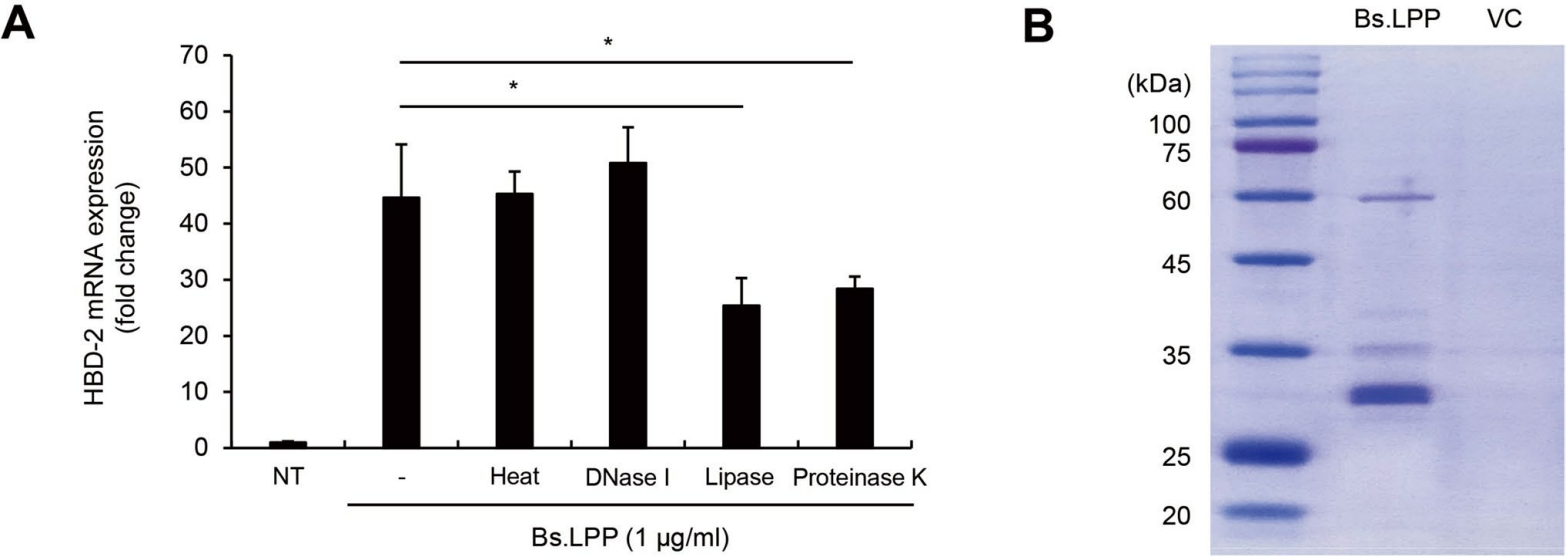
Identification of Bs.LPP candidates. **A** Caco-2 cells (3 $\times$ 10^5^ cells/well) were treated with 1 μg/ml of Bs.LPPs treated with heat, DNase I, LPP lipase, or proteinase K. After treatment for 6 h, total RNA was isolated and the mRNA expression of HBD-2 was measured using real-time RT-PCR. **B** Bs.LPP was visualized with Coomassie blue staining. All results are expressed as the mean $\pm$ standard deviation (S.D.) of triplicate samples. Asterisks (*) indicate statistically significant differences (*P* < 0.05) compared with the appropriate control. NT, non-treatment; VC, vehicle control

To determine whether the induction of HBD-2 was due to impurities in the extract, LPP from *B. subtilis* was treated with heat, DNase I, LPP lipase, or proteinase K. As a result, LPP treated with lipase or proteinase K decreased the mRNA expression of HBD-2, whereas neither heat nor DNase I treatment reduced it (Fig. 7A). These data indicate that LPP is the primary factor responsible for HBD-2 induction and that both the protein and lipid moieties of LPP are important for HBD-2 mRNA upregulation in Caco-2 cells. Because it has been well documented that *B. subtilis* possesses at least 63 functionally distinct LPPs [32], we aimed to identify the LPP responsible for inducing HBD-2. LPP extract from *B. subtilis* was separated on a 10% SDS-PAGE gel. Coomassie blue staining analysis showed that the *B. subtilis* LPPs are about 60 kDa or 30 to 35 kDa (Fig. 7B). In addition, the protein components in the extract were analyzed using Q Exactive Plus mass spectrometry. Information about the putative *B. subtilis* LPP candidates was selected by comparison with known *B. subtilis* LPP sequences, as explained in the Material and Method section. As a result of that analysis, 35 putative LPPs in the LPP were identified (Table 2). Of note, most LPPs were about 30 kDa, except for the OppA protein, which was 61 kDa. This result is consistent with previous data showing that the strongest protein band was near 30 kDa (Fig. 7B). Methionine-binding LPP had the highest molecular percentage among the identified LPPs. Collectively, these data indicate that the putative *B. subtilis* LPP candidates contribute to the induction of HBD-2 in human IECs.

**Table 2:** Identification of LPPs in Bs.LPP extract

**No.**	**Protein (function)**	*Mol (%)	**Size (kDa)**
1	Methionine-binding lipoprotein (methionine ABC transporter)	8.1940	30.393
2	Foldase protein PrsA (protein folding)	1.6382	32.547
3	Arginine-binding extracellular protein ArtP (arginine ABC transporter)	1.1463	28.409
4	L-cystine-binding protein TcyA (cystine and diaminopimelate ABC transporter)	1.0049	29.553
5	Manganese-binding lipoprotein MntA (manganese ABC transporter)	0.7231	33.454
6	Fe (3^+^)-citrate-binding protein YfmC (iron/citrate ABC transporter)	0.3798	35.113
7	Iron-uptake system-binding protein (iron ABC transporter)	0.3747	35.143
8	Putative carboxypeptidase YodJ (cell wall synthesis)	0.3616	30.892
9	Probable siderophore-binding lipoprotein YfiY (siderophore ABC transporter)	0.2222	36.339
10	Oligopeptide-binding protein OppA (oligopeptide ABC transporter)	0.1666	61.543
11	Petrobactin-binding protein YclQ (petrobactin ABC transporter)	0.1616	34.827
12	Quinol oxidase subunit 2 (respiration)	0.1586	36.316
13	Ribose import binding protein RbsB (ribose ABC transporter)	0.1111	32.264
14	Probable amino-acid-binding protein YxeM (S-(2-succino)cysteine ABC transporter)	0.1050	29.349
15	Putative ABC transporter substrate-binding lipoprotein YhfQ (iron/citrate ABC transporter)	0.0808	35.524
16	Iron (3^+^)-hydroxamate-binding protein YxeB (hydroxamate siderophore ABC transporter)	0.0808	35.541
17	Putative lipoprotein YerB (unknown)	0.0778	37.117
18	Probable ABC transporter extracellular-binding protein YckB (unknown)	0.0697	31.757
19	Uncharacterized protein YtkA (assembly of the CuA center in Cytochrome caa3)	0.0677	15.904
20	Iron (3^+^)-hydroxamate-binding protein FhuD (hydroxamate siderophore ABC transporter)	0.0626	34.462
21	Uncharacterized lipoprotein YcdA (required for swarming motility)	0.0535	39.213
22	Phosphate-binding protein PstS (phosphate ABC transporter)	0.0465	31.721
23	Uncharacterized protein YdhK (stress protein)	0.0444	22.519
24	High-affinity zinc uptake system binding-protein ZnuA (zinc ABC transporter)	0.0434	35.752
25	Cytochrome c oxidase subunit 2 (respiration)	0.0384	40.354
26	Putative ABC transporter substrate-binding lipoprotein YvgL (molybdenum transporter)	0.0343	28.395
27	Penicillin-binding protein 3	0.0333	74.531
28	Glycine betaine-binding protein OpuAC (glycine betaine and arsenobetaine ABC transporter)	0.0293	32.251
29	Uncharacterized ABC transporter substrate-binding lipoprotein YvrC (cobalamin ABC transporter)	0.0273	34.275
30	SCO1 protein homolog (assembly of the CuA center in Cytochrome caa3)	0.0212	21.932
31	Uncharacterized lipoprotein YjhA (unknown)	0.0192	24.020
32	D-Alanyl-D-alanine carboxypeptidase DacA (penicillin-binding protein 5)	0.0192	48.777
33	Putative lipoprotein YvcA (complex colony development)	0.0172	28.082
34	Probable iron uptake system component EfeM	0.0101	42.941
35	Uncharacterized lipoprotein YerH (unknown)	0.0101	44.546

Mol (%) means molecule percentages in an extract add

## Discussion

**Fig. 8:**
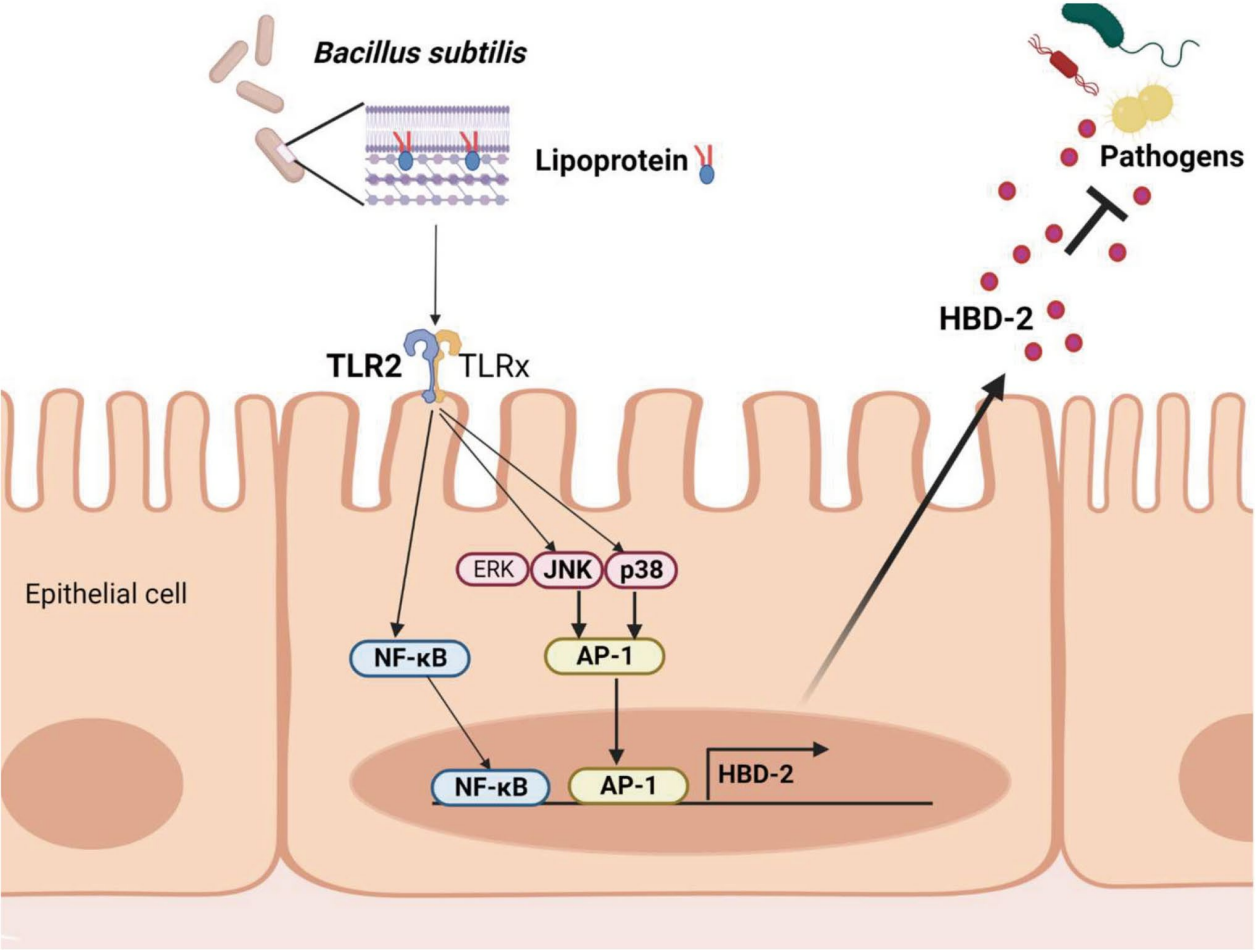
Schematic illustration of the proposed action mechanism

*B. subtilis* is known to be safe for use as a food ingredient or additive and to provide protective effects against GI diseases. Because it was reported that *B. subtilis* contributes to AMP upregulation in the human GI tract [33], understanding the effects of *B. subtilis* and its components on AMP production by human IECs is important. In this study, we have shown that *B. subtilis* induces HBD-2 and its LPP turned out to be one of the major cell wall components responsible for HBD-2 induction. LPP from *B. subtilis* induced HBD-2 expression through TLR2-mediated JNK/p38 MAP kinase/AP-1 and NF-κB pathways and HBD-2 secreted upon LPP stimulation efficiently inhibited the growth of bacterial pathogens (Fig. 8). Collectively, this study suggests that LPP from *B. subtilis* could be a therapeutic agent for protecting the intestinal epithelium.

This study has demonstrated that HBD-2, but not HBD-1 or HBD-3, is induced by *B. subtilis* stimulation. It is concordant with a previous report suggesting that the expression of HBD-2 is increased by bacterial stimulation, whereas HBD-1 production in human IECs is constant [10]. In line with our results, several previous reports have also demonstrated that probiotics can induce HBD-2 production. For instance, treatment with *Escherichia coli* Nissle 1917 or various *Lactobacillus* bacteria increases HBD-2 gene expression, but not HBD-1 expression [34]. In addition, both live and heat-killed *Lactobacillus* species increase the mRNA expression of HBD-2 [35] and probiotic *Bacillus clausii* treatment induces HBD-2 synthesis in human IECs [36]. Those previous reports support our results that probiotics, including *B. subtilis*, can induce AMP production, especially HBD-2 production, protecting the intestinal epithelium against various pathogens.

We identified LPP as a major molecule responsible for the upregulation of HBD-2, rather than other MAMPs of *B. subtilis* such as LTA or PGN. This observation is in accordance with previous reports demonstrating that macrophage-activating lipopeptide-2, a synthetic molecule that mimics the LPP of *Mycoplasma fermentans*, and *Staphylococcus epidermidis* LPP enhanced the expression of HBD-2 in human epidermal keratinocytes [37, 38]. On the other hand, contrary to our findings, several studies identified LTA or PGN as a major molecule responsible for AMP upregulation [18, 39]. One possible explanation for this opposite result might be unexpected contamination of commercial LTA and PGN [40, 41]. In addition, LTA and PGN are known to possess lower immunostimulatory activity than LPP [42]. Therefore, given that those previous studies did not consider the effects of LPP on AMP expression, it can be postulated that the upregulation of AMPs by LTA or PGN could be due to other molecules. On the other hand, TLR2 and NOD2 can synergize to induce high immune responses [43], so it could be possible that other MAMPs work together with LPP to increase AMP production. Collectively, our results suggest that LPP could be a major molecule of Gram-positive bacteria responsible for AMP upregulation.

It has been reported that LPPs can be released during bacterial growth and synthetic bacterial lipopeptide (Pam3CSK4)-induced TLR2 activation leads to AMP production [44, 45]. *B. subtilis* LPP is known as N-acetyl lipoprotein containing N-acetyl-S-diacyl-glyceryl-cysteine and its lipid chain interacts with the TLR2/6 heterodimer [46]. In the present study, *B. subtilis* LPPs induced the increase of HBD-2 expression through TLR2 pathway. Concordantly, S-layer proteins of *Lactobacillus* species induce HBD-2 production via a TLR2 signaling pathway [47]. Also, Pam3CSK4 treatment increases HBD-2 gene expression through TLR2 signaling in human corneal epithelial cells and lung epithelial cells [45]. Notably, we observed that only apical treatment of LPP caused HBD-2 secretion in differentiated Caco-2 cells, suggesting that apical TLR2 recognizes *B. subtilis* LPP and then activates the downstream signaling pathway. In the intestine, TLR2 is expressed at the apical side of differentiated IECs [48] and *B. subtilis* LPPs are known to be recognized by TLR2 [27]. Therefore, given that most microbes are located in the lumen of the intestine, it is likely that TLR compartmentalization is needed to enable immediate and proper antimicrobial responses in the intestine.

In this study, HBD-2 produced by LPP-treated Caco-2 cells inhibited the growth of *S. aureus* and showed a moderate antigrowth effect on *B. cereus*. This differential inhibitory effect could be due to their susceptibility to HBD-2. A previous study revealed that the effective concentration (EC_50_) value of HBD-2 for *S. aureus* was 7.6 μg/ml [49]. Furthermore, *B. cereus* is more susceptible to HBD-2 than to HBD-1 or HBD-3, and the EC_50_ value of HBD-2 for *B. cereus* was 22 μg/ml [50], concordant with our present result demonstrating that HBD-2 induced by LPP treatment more efficiently attenuated the growth of *S. aureus* than *B. cereus*. Although Caco-2 cells release HBD-2 at a maximum concentration of 20 pg/ml, which seems extremely low to show antibacterial activity against both bacteria, the culture supernatant efficiently inhibited the growth of both *B. cereus* and *S. aureus*. Considering that AMPs can trigger further inflammatory responses via their chemotactic activity [6], it is possible that the small amounts of AMPs induced by LPP treatment are sufficient to disable the action of the pathogens. In addition, It has been reported that *B. subtilis* modulates the gut microbiota and blood metabolic profile and probiotics enhance the tight junction proteins of IECs against pathogens [51, 52]. Collectively, both *B. subtilis* and its LPPs likely contribute to the protection of the GI tract against pathogenic bacteria.

In this study, 35 LPPs of *B. subtilis* were identified as candidates that might contribute to HBD-2 upregulation, and most of them were annotated as ABC transporters, responsible for the translocation of proteins and metal ions. On the other hand, they are also recognized for their impact on various aspects of bacterial virulence, including multidrug resistance, adhesion, spore formation, and biofilm formation [53]. Thus, *B. subtilis* LPPs are thought to improve the gut environment by influencing bacterial growth. That finding is in accordance with a previous report showing that *B. subtilis* LPPs predominantly function as transporters [32]. Interestingly, iron uptake transporters, such as the SitC of *S. aureus*, have been reported as TLR2 ligands [54], suggesting that iron-regulated LPPs can act as TLR2 ligands. Moreover, ABC transporters such as MntA, YfmC, YclQ, and OppA are known to be recognized by TLR2 receptors [46, 55]. Although *B. subtilis* ABC transporters and their association with TLR2 receptors are not fully understood, the previous studies support our hypothesis that the identified LPPs contribute to the induction of HBD-2 expression, presumably through TLR2 recognition. Nevertheless, further study is required to clarify which *B. subtilis* LPP is primarily responsible for HBD-2 production.

## Conclusion

In this study, we demonstrated that *B. subtilis* efficiently induces HBD-2 expression in the human IECs. Especially, we have identified a key molecule, *B. subtilis* LPP, that might be responsible for the induction of HBD-2. TLR2-mediated JNK/p38 MAP kinase/AP-1 and NF-κB pathways were critical for the *B. subtilis* LPP-induced HBD-2 induction. Also, *B. subtilis* LPP efficiently inhibited the growth of bacterial pathogens. Collectively, although further in vivo experiments are necessary, this study has identified a major cell wall component of *B. subtilis* that plays crucial roles in protecting intestinal health.

## Data Availability

The data presented in this study are available on request from the corresponding author.

## References

[1] Lozupone CA, Stombaugh JI, Gordon JI, Jansson JK, Knight R (2012) Diversity, stability and resilience of the human gut microbiota. Nature 489(7415):220–230. 10.1038/nature11550 22972295 10.1038/nature11550 PMC3577372

[2] Elmentaite R, Kumasaka N, Roberts K, Fleming A, Dann E, King HW et al (2021) Cells of the human intestinal tract mapped across space and time. Nature 597(7875):250–255. 10.1038/s41586-021-03852-1 34497389 10.1038/s41586-021-03852-1 PMC8426186

[3] Peterson LW, Artis D (2014) Intestinal epithelial cells: regulators of barrier function and immune homeostasis. Nat Rev Immunol 14(3):141–153. 10.1038/nri3608 24566914 10.1038/nri3608

[4] Price AE, Shamardani K, Lugo KA, Deguine J, Roberts AW, Lee BL et al (2018) A map of Toll-like receptor expression in the intestinal epithelium reveals distinct spatial, cell type-specific, and temporal patterns. Immunity 49(3):560–575 e6. 10.1016/j.immuni.2018.07.016 30170812 10.1016/j.immuni.2018.07.016 PMC6152941

[5] Yiu JH, Dorweiler B, Woo CW (2017) Interaction between gut microbiota and toll-like receptor: from immunity to metabolism. J Mol Med (Berl) 95(1):13–20. 10.1007/s00109-016-1474-4 27639584 10.1007/s00109-016-1474-4 PMC5225216

[6] Muniz LR, Knosp C, Yeretssian G (2012) Intestinal antimicrobial peptides during homeostasis, infection, and disease. Front Immunol 3:310. 10.3389/fimmu.2012.00310 23087688 10.3389/fimmu.2012.00310 PMC3466489

[7] Ganz T (2003) Defensins: antimicrobial peptides of innate immunity. Nat Rev Immunol 3(9):710–720. 10.1038/nri1180 12949495 10.1038/nri1180

[8] Ghosh SK, McCormick TS, Weinberg A (2019) Human beta defensins and cancer: contradictions and common ground. Front Oncol 9:341. 10.3389/fonc.2019.00341 31131258 10.3389/fonc.2019.00341 PMC6509205

[9] Cobo ER, Chadee K (2013) Antimicrobial human beta-defensins in the colon and their role in infectious and non-infectious diseases. Pathogens 2(1):177–192. 10.3390/pathogens2010177 25436887 10.3390/pathogens2010177 PMC4235710

[10] O’Neil DA, Porter EM, Elewaut D, Anderson GM, Eckmann L, Ganz T et al (1999) Expression and regulation of the human beta-defensins hBD-1 and hBD-2 in intestinal epithelium. J Immunol 163(12):6718–6724 10586069

[11] Cieslik M, Baginska N, Gorski A, Jonczyk-Matysiak E (2021) Human beta-defensin 2 and its postulated role in modulation of the immune response. Cells 10(11). 10.3390/cells10112991 10.3390/cells10112991 PMC8616480 34831214

[12] Aldhous MC, Noble CL, Satsangi J (2009) Dysregulation of human beta-defensin-2 protein in inflammatory bowel disease. PLoS ONE 4(7):e6285. 10.1371/journal.pone.0006285 19617917 10.1371/journal.pone.0006285 PMC2708916

[13] Earl AM, Losick R, Kolter R (2008) Ecology and genomics of *Bacillus subtilis*. Trends Microbiol 16(6):269–275. 10.1016/j.tim.2008.03.004 18467096 10.1016/j.tim.2008.03.004 PMC2819312

[14] Sewalt V, Shanahan D, Gregg L, La Marta J, Carrillo R (2016) The generally recognized as safe (GRAS) process for industrial microbial enzymes. Ind Biotechnol 12(5):295–302. 10.1089/ind.2016.0011

[15] Su Y, Liu C, Fang H, Zhang D (2020) *Bacillus subtilis*: a universal cell factory for industry, agriculture, biomaterials and medicine. Microb Cell Fact 19(1):173. 10.1186/s12934-020-01436-8 32883293 10.1186/s12934-020-01436-8 PMC7650271

[16] Gu MJ, Song SK, Park SM, Lee IK, Yun CH (2014) *Bacillus subtilis* protects porcine intestinal barrier from deoxynivalenol via improved zonula occludens-1 expression. Asian-Australas J Anim Sci 27(4):580–586. 10.5713/ajas.2013.13744 25049991 10.5713/ajas.2013.13744 PMC4093535

[17] Zou XY, Zhang M, Tu WJ, Zhang Q, Jin ML, Fang RD et al (2022) *Bacillus subtilis* inhibits intestinal inflammation and oxidative stress by regulating gut flora and related metabolites in laying hens. Animal 16(3):100474. 10.1016/j.animal.2022.100474 35220172 10.1016/j.animal.2022.100474

[18] Hou Q, Jia J, Lin J, Zhu L, Xie S, Yu Q et al (2022) *Bacillus subtilis* programs the differentiation of intestinal secretory lineages to inhibit Salmonella infection. Cell Rep 40(13):111416. 10.1016/j.celrep.2022.111416 36170821 10.1016/j.celrep.2022.111416

[19] Mazkour S, Shekarforoush SS, Basiri S, Namazi F, Zarei-Kordshouli F (2022) Protective effects of oral administration of mixed probiotic spores of *Bacillus subtilis* and *Bacillus coagulans* on gut microbiota changes and intestinal and liver damage of rats infected with Salmonella Typhimurium. J Food Saf 42(4):e12981. 10.1111/jfs.12981

[20] Caulier S, Nannan C, Gillis A, Licciardi F, Bragard C, Mahillon J (2019) Overview of the antimicrobial compounds produced by members of the *Bacillus subtilis* group. Front Microbiol 10:302. 10.3389/fmicb.2019.00302 30873135 10.3389/fmicb.2019.00302 PMC6401651

[21] Lee D, Im J, Park DH, Jeong S, Park M, Yoon S et al (2021) *Lactobacillus plantarum* lipoteichoic acids possess strain-specific regulatory effects on the biofilm formation of dental pathogenic bacteria. Front Microbiol 12:758161. 10.3389/fmicb.2021.758161 34867884 10.3389/fmicb.2021.758161 PMC8636137

[22] Han SH, Kim JH, Martin M, Michalek SM, Nahm MH (2003) Pneumococcal lipoteichoic acid (LTA) is not as potent as *staphylococcal* LTA in stimulating Toll-like receptor 2. Infect Immun 71(10):5541–5548. 10.1128/IAI.71.10.5541-5548.2003 14500472 10.1128/IAI.71.10.5541-5548.2003 PMC201083

[23] Baik JE, Jang YO, Kang SS, Cho K, Yun CH, Han SH (2015) Differential profiles of gastrointestinal proteins interacting with peptidoglycans from *Lactobacillus plantarum* and *Staphylococcus aureus*. Mol Immunol 65(1):77–85. 10.1016/j.molimm.2015.01.007 25647716 10.1016/j.molimm.2015.01.007

[24] Kim NJ, Ahn KB, Jeon JH, Yun CH, Finlay BB, Han SH (2015) Lipoprotein in the cell wall of *Staphylococcus aureus* is a major inducer of nitric oxide production in murine macrophages. Mol Immunol 65(1):17–24. 10.1016/j.molimm.2014.12.016 25600878 10.1016/j.molimm.2014.12.016

[25] Gao P, Duan W, Shi H, Wang Q (2023) Silencing circPalm2 inhibits sepsis-induced acute lung injury by sponging miR-376b-3p and targeting MAP3K1. Toxicol Res 39(2):275–294. 10.1007/s43188-022-00169-7 37008689 10.1007/s43188-022-00169-7 PMC10050541

[26] Pedreira T, Elfmann C, Stulke J (2022) The current state of SubtiWiki, the database for the model organism *Bacillus subtilis*. Nucleic Acids Res 50(D1):D875–D882. 10.1093/nar/gkab943 34664671 10.1093/nar/gkab943 PMC8728116

[27] Hashimoto M, Tawaratsumida K, Kariya H, Aoyama K, Tamura T, Suda Y (2006) Lipoprotein is a predominant Toll-like receptor 2 ligand in *Staphylococcus aureus* cell wall components. Int Immunol 18(2):355–362. 10.1093/intimm/dxh374 16373361 10.1093/intimm/dxh374

[28] Kawai T, Akira S (2007) Signaling to NF-kappaB by Toll-like receptors. Trends Mol Med 13(11):460–469. 10.1016/j.molmed.2007.09.002 18029230 10.1016/j.molmed.2007.09.002

[29] Sambuy Y, De Angelis I, Ranaldi G, Scarino ML, Stammati A, Zucco F (2005) The Caco-2 cell line as a model of the intestinal barrier: influence of cell and culture-related factors on Caco-2 cell functional characteristics. Cell Biol Toxicol 21(1):1–26. 10.1007/s10565-005-0085-6 15868485 10.1007/s10565-005-0085-6

[30] Tewari A, Abdullah S (2015) *Bacillus cereus* food poisoning: international and Indian perspective. J Food Sci Technol 52(5):2500–2511. 10.1007/s13197-014-1344-4 25892750 10.1007/s13197-014-1344-4 PMC4397285

[31] Kwak YK, Vikstrom E, Magnusson KE, Vecsey-Semjen B, Colque-Navarro P, Mollby R (2012) The *Staphylococcus aureus* alpha-toxin perturbs the barrier function in Caco-2 epithelial cell monolayers by altering junctional integrity. Infect Immun 80(5):1670–1680. 10.1128/IAI.00001-12 22354024 10.1128/IAI.00001-12 PMC3347457

[32] Nguyen MT, Matsuo M, Niemann S, Herrmann M, Gotz F (2020) Lipoproteins in Gram-positive bacteria: abundance, function, fitness. Front Microbiol 11:582582. 10.3389/fmicb.2020.582582 33042100 10.3389/fmicb.2020.582582 PMC7530257

[33] Pahumunto N, Dahlen G, Teanpaisan R (2021) Evaluation of potential probiotic properties of *Lactobacillus* and *Bacillus* strains derived from various sources for their potential use in swine feeding. Probiotics Antimicrob Proteins. 10.1007/s12602-021-09861-w 34665429 10.1007/s12602-021-09861-w

[34] Wehkamp J, Harder J, Wehkamp K, Wehkamp-von Meissner B, Schlee M, Enders C et al (2004) NF-kappaB- and AP-1-mediated induction of human beta defensin-2 in intestinal epithelial cells by *Escherichia coli* Nissle 1917: a novel effect of a probiotic bacterium. Infect Immun 72(10):5750–5758. 10.1128/IAI.72.10.5750-5758.2004 15385474 10.1128/IAI.72.10.5750-5758.2004 PMC517557

[35] Habil N, Abate W, Beal J, Foey AD (2014) Heat-killed probiotic bacteria differentially regulate colonic epithelial cell production of human beta-defensin-2: dependence on inflammatory cytokines. Benef Microbes 5(4):483–495. 10.3920/BM2013.0061 25116382 10.3920/BM2013.0061

[36] Paparo L, Tripodi L, Bruno C, Pisapia L, Damiano C, Pastore L et al (2020) Protective action of *Bacillus clausii* probiotic strains in an in vitro model of Rotavirus infection. Sci Rep 10(1):12636. 10.1038/s41598-020-69533-7 32724066 10.1038/s41598-020-69533-7 PMC7387476

[37] Buchau AS, Schauber J, Hultsch T, Stuetz A, Gallo RL (2008) Pimecrolimus enhances TLR2/6-induced expression of antimicrobial peptides in keratinocytes. J Invest Dermatol 128(11):2646–2654. 10.1038/jid.2008.135 18496569 10.1038/jid.2008.135 PMC2639780

[38] Li D, Lei H, Li Z, Li H, Wang Y, Lai Y (2013) A novel lipopeptide from skin commensal activates TLR2/CD36-p38 MAPK signaling to increase antibacterial defense against bacterial infection. PLoS ONE 8(3):e58288. 10.1371/journal.pone.0058288 23472173 10.1371/journal.pone.0058288 PMC3589260

[39] Wang X, Zhang Z, Louboutin JP, Moser C, Weiner DJ, Wilson JM (2003) Airway epithelia regulate expression of human beta-defensin 2 through Toll-like receptor 2. FASEB J 17(12):1727–1729. 10.1096/fj.02-0616fje 12958190 10.1096/fj.02-0616fje

[40] Li H, Nooh MM, Kotb M, Re F (2008) Commercial peptidoglycan preparations are contaminated with superantigen-like activity that stimulates IL-17 production. J Leukoc Biol 83(2):409–418. 10.1189/jlb.0807588 17991761 10.1189/jlb.0807588

[41] Hashimoto M, Furuyashiki M, Kaseya R, Fukada Y, Akimaru M, Aoyama K et al (2007) Evidence of immunostimulating lipoprotein existing in the natural lipoteichoic acid fraction. Infect Immun 75(4):1926–1932. 10.1128/IAI.02083-05 17283098 10.1128/IAI.02083-05 PMC1865665

[42] Kang SS, Noh SY, Park OJ, Yun CH, Han SH (2015) *Staphylococcus aureus* induces IL-8 expression through its lipoproteins in the human intestinal epithelial cell, Caco-2. Cytokine 75(1):174–180. 10.1016/j.cyto.2015.04.017 25982554 10.1016/j.cyto.2015.04.017

[43] Ahn KB, Jeon JH, Baik JE, Park OJ, Kang SS, Yun CH et al (2014) Muramyl dipeptide potentiates *staphylococcal* lipoteichoic acid induction of cyclooxygenase-2 expression in macrophages. Microbes Infect 16(2):153–160. 10.1016/j.micinf.2013.10.018 24211871 10.1016/j.micinf.2013.10.018

[44] Zhang H, Niesel DW, Peterson JW, Klimpel GR (1998) Lipoprotein release by bacteria: potential factor in bacterial pathogenesis. Infect Immun 66(11):5196–5201. 10.1128/IAI.66.11.5196-5201.1998 9784522 10.1128/iai.66.11.5196-5201.1998 PMC108648

[45] Kumar A, Zhang J, Yu FS (2006) Toll-like receptor 2-mediated expression of beta-defensin-2 in human corneal epithelial cells. Microbes Infect 8(2):380–389. 10.1016/j.micinf.2005.07.006 16242370 10.1016/j.micinf.2005.07.006 PMC2666383

[46] Kurokawa K, Ryu KH, Ichikawa R, Masuda A, Kim MS, Lee H et al (2012) Novel bacterial lipoprotein structures conserved in low-GC content gram-positive bacteria are recognized by Toll-like receptor 2. J Biol Chem 287(16):13170–13181. 10.1074/jbc.M111.292235 22303020 10.1074/jbc.M111.292235 PMC3339964

[47] Kobatake E, Kabuki T (2019) S-Layer Protein of *Lactobacillus helveticus* SBT2171 promotes human beta-defensin 2 expression via TLR2-JNK signaling. Front Microbiol 10:2414. 10.3389/fmicb.2019.02414 31681252 10.3389/fmicb.2019.02414 PMC6813279

[48] Cario E, Brown D, McKee M, Lynch-Devaney K, Gerken G, Podolsky DK (2002) Commensal-associated molecular patterns induce selective toll-like receptor-trafficking from apical membrane to cytoplasmic compartments in polarized intestinal epithelium. Am J Pathol 160(1):165–173. 10.1016/S0002-9440(10)64360-X 11786410 10.1016/S0002-9440(10)64360-X PMC1867149

[49] Chen X, Niyonsaba F, Ushio H, Okuda D, Nagaoka I, Ikeda S et al (2005) Synergistic effect of antibacterial agents human beta-defensins, cathelicidin LL-37 and lysozyme against *Staphylococcus aureus* and *Escherichia coli*. J Dermatol Sci 40(2):123–132. 10.1016/j.jdermsci.2005.03.014 15963694 10.1016/j.jdermsci.2005.03.014

[50] Yadava P, Zhang C, Sun J, Hughes JA (2006) Antimicrobial activities of human beta-defensins against *Bacillus* species. Int J Antimicrob Agents 28(2):132–137. 10.1016/j.ijantimicag.2006.02.021 16797165 10.1016/j.ijantimicag.2006.02.021

[51] Li G, Tong Y, Xiao Y, Huang S, Zhao T, Xia X (2023) Probiotic *Bacillus subtilis* contributes to the modulation of gut microbiota and blood metabolic profile of hosts. Comp Biochem Physiol C Toxicol Pharmacol 272:109712. 10.1016/j.cbpc.2023.109712 37544638 10.1016/j.cbpc.2023.109712

[52] Blackwood BP, Yuan CY, Wood DR, Nicolas JD, Grothaus JS, Hunter CJ (2017) Probiotic *Lactobacillus* species strengthen intestinal barrier function and tight junction integrity in experimental necrotizing enterocolitis. J Probiotics Health 5(1). 10.4172/2329-8901.1000159 10.4172/2329-8901.1000159 PMC5475283 28638850

[53] Akhtar AA, Turner DP (2022) The role of bacterial ATP-binding cassette (ABC) transporters in pathogenesis and virulence: therapeutic and vaccine potential. Microb Pathog 171:105734. 10.1016/j.micpath.2022.105734 36007845 10.1016/j.micpath.2022.105734

[54] Muller P, Muller-Anstett M, Wagener J, Gao Q, Kaesler S, Schaller M et al (2010) The *Staphylococcus aureus* lipoprotein SitC colocalizes with Toll-like receptor 2 (TLR2) in murine keratinocytes and elicits intracellular TLR2 accumulation. Infect Immun 78(10):4243–4250. 10.1128/IAI.00538-10 20679445 10.1128/IAI.00538-10 PMC2950364

[55] Kurokawa K, Lee H, Roh KB, Asanuma M, Kim YS, Nakayama H et al (2009) The triacylated ATP binding cluster transporter substrate-binding lipoprotein of *Staphylococcus aureus* functions as a native ligand for Toll-like receptor 2. J Biol Chem 284(13):8406–8411. 10.1074/jbc.M809618200 19139093 10.1074/jbc.M809618200 PMC2659198

